# Botulinum Toxin Treatment in Musician’s Dystonia: Long-Term-Effects and Influencing Factors

**DOI:** 10.3390/toxins18070317

**Published:** 2026-07-21

**Authors:** Till-Alexander Plinkert, Johanna Doll-Lee, Edoardo Passarotto, Christos I. Ioannou, Eckart Altenmüller, André Lee

**Affiliations:** 1 Institute for Music Physiology and Musicians’ Medicine, University of Music, Drama and Media Hannover, 30175 Hannover, Germany; 2 Department of Neurology, Hanover Medical School, 30625 Hannover, Germany; 3 Center for System Neurosciences, 30559 Hannover, Germany; 4 Department Neuroscience, University of Padova, 35122 Padova, Italy; 5 Department of Rehabilitation Sciences, Cyprus University of Technology, 3036 Limassol, Cyprus; 6 Department of Neurology, TUM Klinikum Rechts der Isar, TUM Medical School, 81675 Munich, Germany

**Keywords:** botulinum toxin, dystonia, musicians’ dystonia

## Abstract

Musician’s Dystonia (MD) is a task-specific movement disorder that occurs while playing an instrument and severely limits a musician’s ability to perform. While currently no causal therapies are available, symptomatic therapies, particularly the injection of botulinum toxin (BoNT), offer a therapeutic option. The present study presents a 30-year longitudinal evaluation of the clinical course of 156 patients with MD treated with BoNT at a specialist outpatient facility. The evaluation took into account known risk factors, such as family history, time to treatment, initial severity of symptoms, gender, and age at the onset of playing an instrument and the onset of symptoms. The study showed that more severe initial symptoms, which entailed a larger number of muscles requiring treatment, predicted a poorer long-term outcome. Furthermore, a longer interval between symptom onset and the commencement of BoNT treatment was also associated with a poorer outcome. These results highlight the importance of rapid diagnosis and the early initiation of BoNT therapy in MD patients.

## 1. Introduction

Musician’s Dystonia (MD) is a task-specific, focal dystonia characterized by involuntary muscle contractions [1,2]. While being painless, they result in a loss or deterioration of fine motor control of the highly trained executive functions required for playing an instrument [3]. The assumed prevalence among professional musicians is around 1% [4], with men being affected up to four times as often as women [3,5]. The average age of onset for MD is around 33 years, often situated at the peak of a musician’s career, which can lead to significant psychosocial repercussions, as the onset coincides with their professional development [3,6,7,8]. Interestingly, women tend to get the disease earlier than men [5]. The prognosis for MD remains ambiguous once established, as evidenced by a long-term study indicating that only 5.6% of patients achieved complete remission, while 81.5% reported subjective improvements with multimodal treatment approaches [8]. Nevertheless, MD imposes substantial limitations on performance, frequently precipitating the end of a musician’s professional activity [4,8,9].

The etiology of MD has not yet been conclusively clarified. Recent research suggests that MD originates not from a singular dysfunctional brain area but is a network disorder comprising functional and structural alterations of several sensorimotor and other cortical areas as well as the basal ganglia and the cerebellum [10,11,12,13,14,15,16,17,18,19,20,21].

Various risk factors have been identified in epidemiological studies, including male gender, instrument type, and temporal and spatial constraints. Other factors include maladaptive practice behavior, as well as psychological and genetic factors [5,22,23,24,25,26]. Although there is currently no causal treatment option, several symptomatic therapies are available, including anticholinergic drugs, retraining, and botulinum toxin (BoNT) injections into the affected body part [8,20,27]. The latter has been shown to be one of the most effective therapies for focal, task-specific dystonia of the upper extremity [28,29].

Previous research indicates that patients diagnosed with MD and exhibiting high initial requirements for BoNT generally necessitate increased doses over time as the condition progresses. This correlation serves as a potential indicator of a poorer clinical prognosis [28]. Additionally, a positive family history of movement disorders has been associated with less favorable outcomes for those affected by MD, highlighting the potential genetic component to this condition [7].

With this background, we propose the following hypotheses:

The outcome parameters in terms of playing ability would be modified by:

1. The age at onset of MD;
2. A positive family history for Movement Disorders;
3. The severity of MD at onset;
4. The symptom-to-treatment interval.
5. Moreover, we hypothesized the following.
6. The clinical course would correlate with the number of muscles initially treated.
7. As women tend to be younger at the onset of MD, previous studies suggested that men seek healthcare later than women [30,31,32]. We finally hypothesized the following.
8. There would be differences between male and female participants in age at onset and time-to-treatment interval.

## 2. Results

Baseline characteristics and sex-stratified analyses are summarized in Table 1. The median age at the time of completing the questionnaire was 52 years. A total of 31 patients (19.9%) had a positive family history of dystonia or another movement disorder. In total, patients in our cohort included 18 different types of instrumentalists and one conductor. In total, 134 (85.9%) were male and 22 (14.1%) were female (female-to-male ratio ≈ 1:6). Patients had started to play their instrument at a median age of 9 years. They recognized the first symptoms of MD at a median age of 35 and received their first BoNT treatment at a median age of 42 years. Subjective playing ability at T0 was 50% and at T1 70%, resulting in a significant median increase of 20% in subjective playing abilities (*p*-value < 0.001) over the observation period (delta_playing_ability).

Treatment-related outcomes during follow-up are summarized in Table 2. In total, 1780 treatment sessions were documented. The median number of sessions per patient was six, receiving a median of 31.9 units (U) across 21 months; injections were administered at a median injection interval of 5.5 months. Each treatment session involved a median of 14.2 U injected into 2–3 target muscles.

While some patients were still undergoing regular treatment at the time of completing the questionnaire, others had received their last BoNT injection some considerable time ago. The median time elapsed since the last injection was 4.0 years (Q1–Q3: 0.2–8.7). Among all participants, 87 reported side effects from BoNT therapy, primarily temporary muscle weakness. Of those who reported side effects, only 10 discontinued BoNT therapy due to adverse effects. Forty-nine participants stopped therapy due to a lack of symptom improvement.

**Figure 1:**
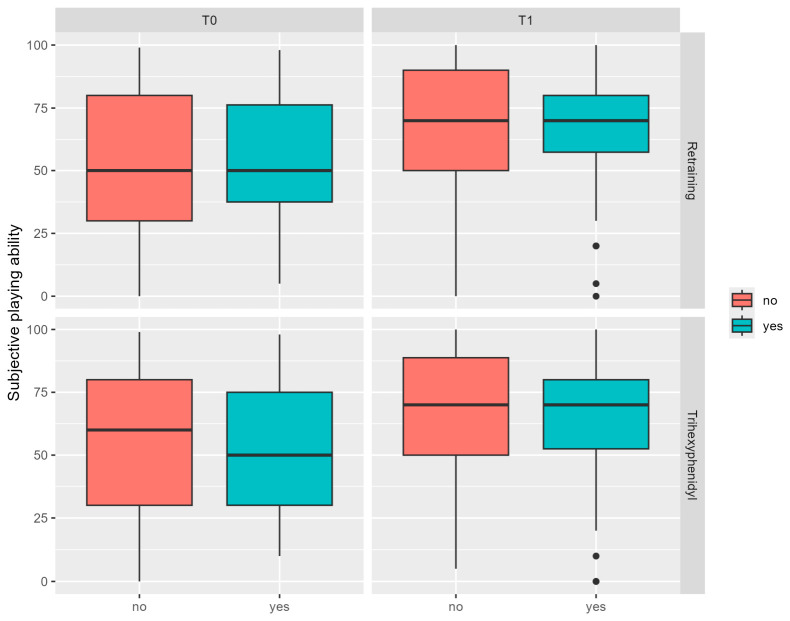
*n* = 154. The figure shows subjective playing ability (%) in patients undergoing concomitant BoNT and pharmacological or retraining treatments, both at T0 and T1. None of the group differences was statistically significant.

To take into account treatments that were performed in addition to BoNT therapy, we investigated differences in subjective playing ability (subjplay) scores associated with treatments concomitant to BoNT injections, namely pharmacological treatments (i.e., trihexyphenidyl, THX) and retraining. The comparison was stratified by time points (i.e., T0 and T1). None of the comparisons between treatment groups reached statistical significance (unpaired *t*-test, *p* > 0.05, Figure 1).

Adverse drug reactions to THX were reported by patients in 55% of cases, with dizziness and autonomic reactions such as palpitations being the most common complaints. Drug intake was discontinued in 81% of cases due to adverse reactions or lack of effectiveness. By contrast, adverse events occurred in just 10% of cases during retraining therapy, specifically acute, activity-induced pain.

**Figure 2:**
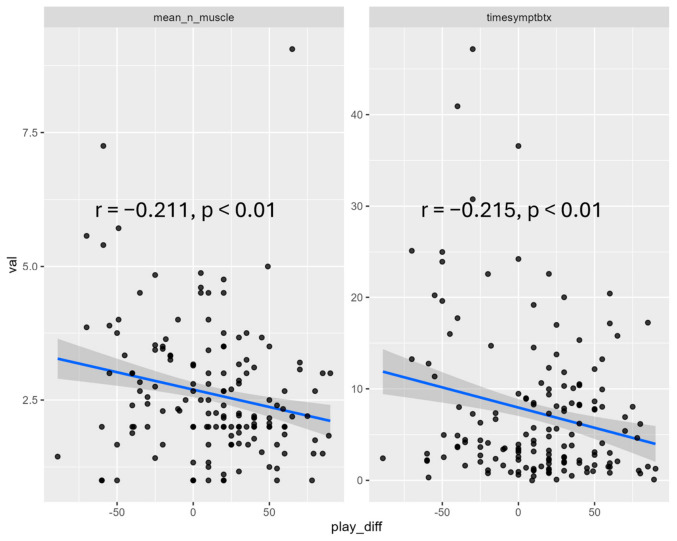
*n* = 154. The figure shows the association between mean_n_muscle, timesympBoNT and delta_subjplay, i.e., the change in subjective playing ability over time. This association was assessed using Pearson’s r coefficients, which were both significant at *p* < 0.01.

A summary of the Bayesian mixed-effects regression model is reported in Table 3. All variables had a Variance Inflation Factor (VIF) < 4, indicating the absence of multicollinearity [31]. The model highlights a substantial effect of *time:mean_n_muscle* and *time:timesymptBoNT*. Thus, the greater the number of injected muscles across BoNT treatments, the lower the improvement in *subjplay* between T0 and T1 (*time:mean_n_muscle*, beta = −8.02, 95% CI [−14.35, −1.85]). Similarly, the larger the distance between MD onset and the beginning of the BoNT treatment, the lower the improvement in *subjplay* over time (*time:timesympBoNT*, beta = −8.37, 95% CI [−14.59, −2.26]) (Figure 2). The effects of the other predictors could not be defined as either positive or negative within a 95% probability. The model explained a substantial amount of variance in the dependent variable, with marginal R2 = 0.165 (i.e., variance explained by fixed effects alone) and conditional R2 = 0.205 (i.e., variance explained by fixed and random effects together).

**Figure 3:**
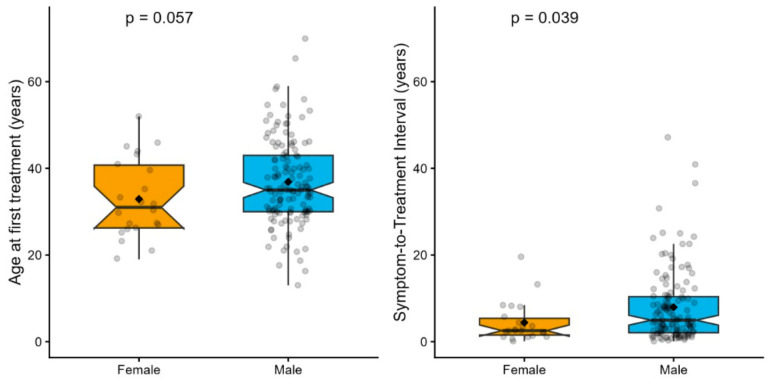
Age at symptom onset (years), age at first treatment (years), and time from symptom onset to first BoNT treatment (years), stratified by sex. Boxplots show the median (horizontal line), interquartile range (box), and 1.5 × IQR whiskers; individual observations are overplotted with gray dots. A black diamond indicates the mean. Sample sizes: males *n* = 134, females *n* = 22. Group comparisons were performed with two-sided Wilcoxon rank-sum tests; exact *p*-values are shown in the panels.

Post hoc, we tested for differences in age at symptom onset and time-to-treatment interval between male and female participants (Table 4, Figure 3). We found that female participants received their first BoNT treatment at a significantly earlier age and that the symptom-to-treatment interval was significantly lower in women (2.6 years) than in male participants (4.9 years).

## 3. Discussion

The aim of this study was to delineate the long-term course of botulinum toxin (BoNT) treatment in Musician’s Dystonia (MD) by examining inter-injection intervals alongside established risk factors and treatment parameters such as age at onset, positive family history, symptom-to-treatment interval, initial BoNT dose, and the number of treated muscles. To our knowledge, this is the first study to integrate these factors within a continuous, multi-decade follow-up (1994–2024), representing the longest observational period of BoNT therapy in MD reported to date.

To control for the potential influence of concomitant therapies, we first compared symptom severity at dystonia onset and at the time of questionnaire completion between patients who had received no additional treatment and those who had undergone concomitant retraining or received concomitant pharmacological treatment with Trihexyphenidyl (THX). No significant differences were observed between these groups, indicating that the observed effects cannot readily be explained by concomitant therapies alone and are therefore likely to be attributable, at least in part, to BoNT treatment.

The median difference in subjectively assessed playing ability between T0 and T1 corresponded to a 20% improvement. Although this result was statistically significant, an improvement of this magnitude may not be sufficient for individual musicians to resume a professional career. Given the substantial interindividual variability, however, it is difficult to draw general conclusions for the entire cohort. Nevertheless, clinical experience indicates that even modest symptom relief, especially when accompanied by compensatory strategies such as adapted fingering techniques or repertoire adjustments, may enable some musicians to continue their professional careers.

We observed a median age at onset of MD of 35 years, consistent with prior work reporting onset in the mid-thirties range [7,8]. Contrary to our initial hypothesis, the age at symptom onset did not significantly influence the clinical outcome. A positive family history of movement disorders was present in approximately 20% of cases, modestly higher than in some prior MD reports [3,7]. Importantly, positive family history was associated with worse outcomes, supporting our second hypothesis. This pattern aligns with prior observations that hereditary or familial factors can lead to a less favorable course in MD [7].

The median interval between first symptoms and first BoNT treatment was 4 years, with occasional delays up to 10 years, underscoring barriers to timely diagnosis and access to expert care for MD. As anticipated in our fourth hypothesis, longer delays between onset and treatment start correlated with worse clinical outcomes. This finding may reflect a cycle in which delayed diagnosis leads to maladaptive practice routines and entrenched motor patterns, reducing the potential for retraining benefits and durable improvement once BoNT therapy is finally started. Early initiation appears to enhance the probability of successful retraining and sustained improvement in playing ability.

Furthermore, we found that the number of muscles initially treated—which is closely linked with the amount of BoNT injected per session-was negatively associated with treatment outcome, confirming our fifth hypothesis. This finding is consistent with the aforementioned study, which likewise reported a negative correlation between the initial amount of BoNT administered and the course of treatment [28]. The number of muscles initially injected with BoNT depends on the extent of dystonia, that is, on how many body parts are affected (e.g., how many fingers are involved). For example, in cases of flexion dystonia affecting a single finger, the corresponding Mm. flexores profundi and superficiales are usually injected, whereas injections into the Mm. lumbricales or interossei are rarely required. Accordingly, it can be assumed that more severe symptoms affecting several fingers necessitate treatment of a greater number of muscles and, consequently, a higher total dose of BoNT. Therefore, it is not surprising that participants with a higher number of treated muscles showed less improvement, which also confirms our third hypothesis.

The median BoNT dose per session in our cohort was 14.2 U, which is consistent with doses previously reported for the treatment of patients with MD [28]. On average, two to three muscles were injected at treatment intervals of 5.5 months. The higher BoNT doses reported in studies focusing on Writer’s Cramp [33,34,35,36], another task-specific focal hand dystonia, are most likely attributable to the fact that musicians with MD require a particularly high level of functional outcome in order to continue practicing their profession. In these patients, the target muscle should only be weakened sufficiently to prevent pronounced involuntary finger flexion while preserving the ability to voluntarily flex the affected finger; excessive weakness must therefore be avoided. In clinical practice, achieving this balance requires careful titration of individual treatment regimens and the use of ultrasound-guided injections.

There was a trend showing that female patients developed MD earlier and sought medical help sooner than male patients. An earlier onset of the disease in women has been described previously and may be related to the comparatively greater influence of genetic factors [5].

Interestingly, women also showed a more favorable treatment outcome. A plausible explanation for this finding is that female patients sought medical care sooner after symptom onset, confirming our sixth hypothesis. In our cohort, an earlier initiation of treatment was associated with better outcomes, suggesting that the superior outcome observed in women may, at least in part, be mediated by shorter delays between symptom onset and treatment initiation. This interpretation is consistent with previous research indicating that women generally make greater use of healthcare services and tend to seek professional medical help earlier than men [37,38,39].

Although the present study does not allow causal conclusions, the findings highlight the potential importance of early recognition and treatment of MD and suggest that delayed healthcare utilization may adversely affect long-term outcomes.

### Limitations

Our study has several limitations that should be considered when interpreting the findings.

First, due to the uneven distribution of instrument types within our cohort, we were unable to adequately control for potential instrument-specific effects. Since the clinical presentation of MD and the functional demands placed on affected musicians may vary across different instruments, such effects cannot be excluded. For the same reason, we were unable to perform statistical analyses for the different BoNT preparations.

Second, treatment outcome was assessed exclusively using subjective measures of playing ability reported by the participants. No objective clinician-rated assessment or expert evaluation of musical performance was available. Consequently, the reported outcomes may have been influenced by individual perceptions and reporting biases.

Third, our analyses focused on patients who had received repeated BoNT treatments, as our aim was to investigate factors related to BoNT therapy, including inter-injection intervals. Patients who had never received BoNT treatment, which applies to all patients with embouchure dystonia, and those who underwent only a single injection were therefore not included, as no inter-injection interval can be determined in the latter group. This limits the generalizability of our findings to the broader population of musicians with MD and may have introduced a selection bias toward patients who continued treatment over time.

## 4. Conclusions

The most clinically relevant finding of this study is that earlier initiation of BoNT therapy was associated with significantly better outcomes in musicians with MD, underscoring the importance of timely diagnosis and treatment. In contrast, neither the age at which an instrument was first played nor the age at disease onset appeared to influence treatment efficacy.

As expected, greater symptom severity at disease onset, likely reflected by the need to treat a larger number of muscles, was associated with a less favorable outcome. Similarly, a positive family history of movement disorders predicted poorer treatment response. We also observed that women sought treatment earlier than men and achieved better outcomes, a finding that may reflect sex-specific differences in healthcare-seeking behavior.

These results highlight the critical importance of reducing delays between symptom onset and treatment initiation. Improving awareness of MD among healthcare professionals, music teachers, and other individuals working closely with musicians may facilitate earlier recognition, prompt referral to specialized centers, and ultimately improve long-term outcomes.

## 5. Materials and Methods

### 5.1. Participants

We retrospectively screened 670 patients with Musician’s Dystonia (MD) treated at our institute. Patients were eligible for inclusion if they had MD affecting the upper limb, were at least 18 years of age, had consented to be contacted for research purposes, and had received at least two botulinum toxin (BoNT) injections at our institute.

We defined a minimum of two BoNT injections as an inclusion criterion for several reasons. The primary reason was clinical: in our experience, the effect of a single BoNT injection is insufficient to reliably assess its therapeutic impact on dystonia. Consequently, the assessment of playing ability after only one injection is of limited interpretability, and conclusions regarding the treatment effect of BoNT remain uncertain.

Second, restricting the analysis to patients who had received at least two injections enabled us to include at least one inter-injection interval in the analysis. This was considered relevant because previous studies have suggested an association between injection intervals and clinical progression.

Finally, all included patients had attended our center on at least two occasions, ensuring that they had received comparable counseling regarding additional therapeutic options. As a general rule, oral trihexyphenidyl (THX) is the initial treatment, if the patients consent, whereas BoNT therapy is introduced when the response to THX is insufficient, or THX is not well tolerated. In addition, retraining therapy is recommended from the outset. Thus, all patients included in the study had the opportunity to consider and, if desired, pursue these additional treatment approaches before starting BoNT therapy.

Of the 670 screened patients, 409 were excluded. Specifically, 250 patients had never received BoNT injections, including patients who were not eligible for BoNT treatment due to certain forms of MD, such as embouchure dystonia. A further 110 patients were excluded because they had received only one BoNT injection at our institute, and 49 patients were excluded because they were younger than 18 years of age or had not consented to being contacted for research purposes. Consequently, 261 patients met the inclusion criteria and were invited to participate in the study.

All included patients received diagnostic evaluation, BoNT treatment, and pharmacological management exclusively at our institute, ensuring consistency of clinical assessment and treatment procedures without variability related to changes in healthcare providers or treatment centers. Retraining therapy was not performed at our institute but could be initiated externally following our recommendation, depending on patient preference.

**Figure 4:**
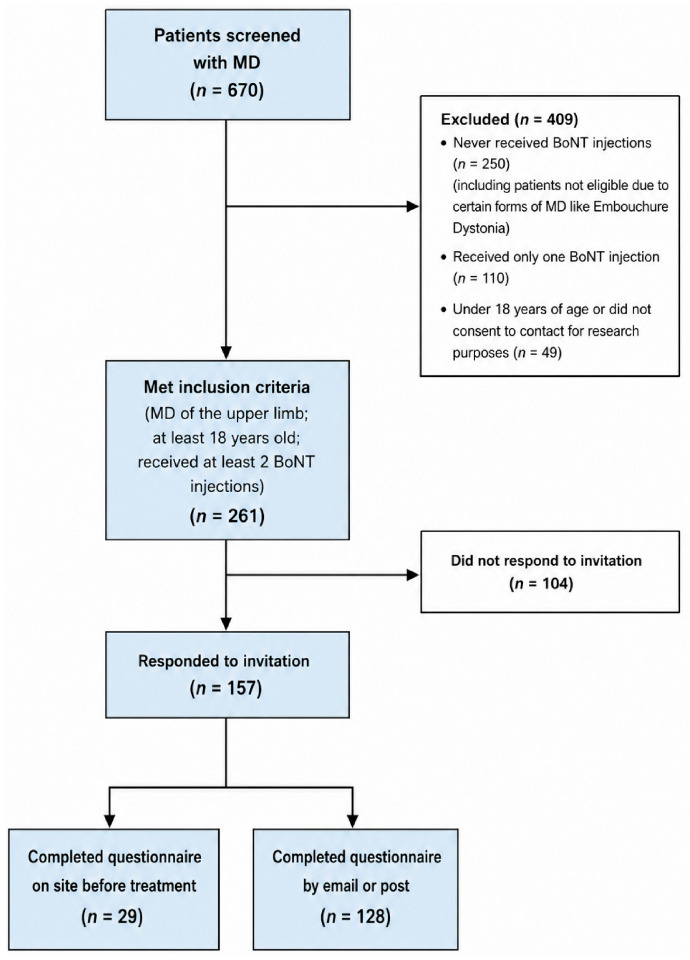
Flowchart of the patient inclusion process.

All eligible patients were invited to take part in the study by completing a questionnaire. Of the 261 contacted patients, 157 (59.77%) responded. Twenty-nine patients completed the questionnaire on site prior to their scheduled BoNT treatment, whereas the remaining participants returned the questionnaire via email or postal mail (Figure 4).

Patients were treated with one of the three botulinum neurotoxin type A (BoNT-A) formulations commercially available in Europe for the management of dystonia: onabotulinumtoxinA, abobotulinumtoxinA, or incobotulinumtoxinA [32].

A total of 100 mouse units (MU) of OnabotulinumtoxinA (ONA; Botox^®^, Abbvie Deutschland GmbH & Co. KG, Wiesbaden, Germany), IncobotulinumtoxinA (INC; Xeomin^®^, Merz Pharma GmbH & Co. KGaA, Frankfurt/Main, Germany), or 500 MU of AbobotulinumtoxinA (ABO; Dysport^®^, Ipsen Pharma GmbH, Ettlingen, Germany) were diluted in 1 mL of sodium chloride solution. Typically, one injection site per muscle was used.

Follow-up treatment sessions were scheduled according to individual clinical needs. During these visits, treatment efficacy and adverse effects were discussed, and BoNT-A dosages were adjusted accordingly. In addition, changes in the number of dystonia-affected fingers led to corresponding adjustments in the number of injected muscles.

For comparability, BoNT-A doses were normalized across preparations, assuming an equivalence ratio of 1 MU of ONA or INC to 3 units of ABO [40].

### 5.2. Questionnaire, Parameters and Follow-Up

Besides demographic aspects, the core part of the questionnaire focused on three main topics:

1. Individual dystonia-related medical history:
  - The onset age of musical education (age_startplay).
  - The age of symptom onset for MD (age_firstsympt).
  - Self-rated playing abilities (subjplay):
  - At T0, immediately after the onset of MD (subjplayearly).
  - At T1, at the time of the investigation (subjplaytoday).
2. Evaluation of BoNT therapy:
  - Treatment progression.
  - Assessment of therapeutic efficacy.
3. Risk factors:
  - Family history of movement disorders (fam_history).

Treatment-related clinical data (TRC) from each BoNT session was recorded continuously in the medical chart over the course of the 30-year follow-up. This included the treatment date (date 1, date 2, …), the type of BoNT preparation used, the mean amount of BoNT injected per session (mean_BoNT), the mean number of muscles treated per session (mean_n_muscles)—used to calculate the relative dose (BoNT_mean_muscle)—and the mean inter-injection interval (mean inter-injection interval). *Delta_subjplay* was computed as the difference between *subjplay* at T1 and *subjplay* at T0.

### 5.3. Statistics

A Bayesian mixed effects regression model was run to identify which variables effectively predict the change in the subjective playing ability between T0 and T1. Thus, *subjplay* was regressed on time, sex, family history, the mean number of muscles treated per session, the duration of the treatment (cum_time), the mean inter-injection interval (mean inter-injection interval), the mean amount of BoNT injected per session, the symptom-to-treatment time interval (timesymptBoNT), the age at which the MD symptoms began, and the age at which music education began. The effect of each predictor on the progression of the disease was assessed in terms of two-way interactions with time. In order to account for interindividual differences in baseline values (i.e., subjective playing ability (*subjplay*) at T0), the model intercept was allowed to vary across patients as a random effect. Given the range of *subjplay* (0–1), the variable was modeled as a beta distribution using the log link function. Time was coded as follows: *subjplay* at T0 = 0 and *subjplay* at T1 = 1. To ensure sex-neutral model estimates, *sex* was coded as females = −0.5 and males = 0.5. Continuous variables were scaled and centered to have M = 0 and SD = 1. In addition, the model was adjusted for playing ability at MD onset by regressing delta_playing_ability on playing_ability at T0. Potential multicollinearity issues between predictors were ruled out by considering the Variance Inflation Factor (VIF) value. The statistical analyses were run using R (Version 4.5). The results report the estimated effects together with 95% credibility intervals (CIs). If the interval does not contain zero, the regressors are assumed to have a substantial effect on the dependent variable with at least a 95% probability. For gender-related analysis, we considered the following dependent variables: the absolute and relative amount of BoNT injected per session, the number of muscles treated per session, and the injection intervals. The independent variables were the severity of MD, the clinical course, the family history, and the time-to-treatment interval.

## Figures and Tables

**Table 1:** Participants’ characteristics.

Variable	*n* (%)	Median (Q1–Q3)
Participants	156	
Female	22 (14.1)	
Family history of movement disorders	31 (19.9)	
Age when completing the form ^1^		52 (40–60)
Age ^1^ at the start of playing		9 (7–13)
Age ^1^ at onset of MD		35 (30–43)
Age ^1^ at first BoNT treatment		42 (35–51)
Subjective playing ability ^2^ at T0		50 (30–73)
Subjective playing ability ^2^ at T1		70 (50–80)
Delta playing ability ^3^		20 (−15–40)
Symptom-to-treatment interval ^1,4^		4 (2–10)
Pharmacological mediation (i.e., trihexiphenidyl)	32 (20.5)	
Retraining	42 (27.2)	

^1^ (years); ^2^ (percentage); ^3^ delta_playing_ability; ^4^ timesymptBoNT.

**Table 2:** Follow-up treatment parameters.

Variable	Median	Q1–Q3
mean_n_muscles	2.3	2.0–3.2
cum_time	25	3–66
mean_BoNT	14.2	9.7–22.9
mean_inter-injection_interval	5.5	3.6–9.5
mean_n_treatments	5	2–11
mean_total_units	33.3	22.6–50.5

Total treatments: *n* = 1780. Abbreviations: mean_n_muscles, mean number of muscles injected per session; cum_time (months), cumulative time under treatment; mean_BoNT (units) is the mean BoNT dose per session; mean_inter-injection_interval (months) is the mean inter-injection interval; mean_n_treatments is the mean number of treatments during follow-up; mean_total_units (mouse units) is the mean total BoNT dose during follow-up.

**Table 3:** Fixed-effect estimates from the Bayesian mixed-effects regression model.

Predictor	b	SE	Lower 95% CI	Upper 95% CI
Intercept	0.118	0.154	−0.182	0.415
time	0.064	0.209	−0.350	0.464
sex	0.072	0.281	−0.480	0.625
**fam_history**	**−0.495**	**0.254**	**−0.992**	**−0.014**
cum_time	−0.027	0.097	−0.219	0.159
mean inter-injection interval	−0.037	0.088	−0.212	0.138
mean_BoNT	−0.089	0.092	−0.267	0.088
mean_n_muscle	0.030	0.094	−0.154	0.218
timesympBoNT	0.098	0.095	−0.087	0.291
age_firstsympt	0.012	0.099	−0.185	0.208
age_startplay	−0.069	0.095	−0.257	0.114
time:sex	0.387	0.380	−0.365	1.141
time:fam_history	0.653	0.348	−0.023	1.308
time:cum_time	0.153	0.135	−0.107	0.419
time:mean inter-injection interval	0.045	0.127	−0.205	0.290
time:mean_BoNT	−0.080	0.122	−0.325	0.158
**time:mean_n_muscle**	**−0.412**	**0.128**	**−0.661**	**−0.164**
**time:timesympBoNT**	**−0.326**	**0.127**	**−0.575**	**−0.079**
time:age_firstsympt	−0.149	0.133	−0.400	0.113
time:age_startplay	0.121	0.131	−0.142	0.369

Notes: N = 154; in the model, intercept values were allowed to vary across patients as random effects; fam_history = family history for movement disorders; mean_n_muscle = number of muscles injected per session; cum_time (months) = cumulative time under treatment; mean_BoNT (units) = mean BoNT dose per muscle per session. mean inter-injection interval = mean inter-injection interval (months) l; timesymptBoNT = time from symptom onset to first BoNT treatment (years); age_firstsympt = age at first symptoms; age_startplay = age at start of instrumental training. Statistically significant values are highlighted in bold.

**Table 4:** Sex-stratified subgroup analysis of key baseline and treatment-timing variables.

Predictor	Male (134)	Female (22)	*p*
age_firstsympt ^1^	35.0 (30.0–43.0)	31.0 (26.3–40.8)	0.057
age ^1^ at first BoNT treatment	42.8(36.6–51.7)	34.6 (27.7–43.5)	0.005 *
timesymptBoNT ^1^	4.9 (2.1–10.4)	2.6 (1.5–5.4)	0.046 *

Values are medians with interquartile ranges (Q1–Q3). Group comparisons were performed with two-sided Wilcoxon rank sum tests. Age_firstsympt = Age at first MD symptoms, timesymptBoNT = time from first symptoms until first treatment with BoNT. * *p* < 0.05; ^1^ years.

## Data Availability

The data presented in this study are available on reasonable request from the corresponding author due to patients’ privacy.

## Abbreviations

The following abbreviations are used in this manuscript:

BoNT	Botulinum toxin
MD	Musician’s Dystonia
PRO	Patient-reported outcome
THX	Trihexyphenidyl
U	Unit
VIF	Variance inflation factor
